# Interleukin-6 in Serum and in Synovial Fluid Enhances the Differentiation between Periprosthetic Joint Infection and Aseptic Loosening

**DOI:** 10.1371/journal.pone.0089045

**Published:** 2014-02-21

**Authors:** Thomas M. Randau, Max J. Friedrich, Matthias D. Wimmer, Ben Reichert, Dominik Kuberra, Birgit Stoffel-Wagner, Andreas Limmer, Dieter C. Wirtz, Sascha Gravius

**Affiliations:** 1 University Clinic of Bonn, Department of Orthopedics and Trauma Surgery, Bonn, Germany; 2 University Clinic of Bonn, Institute for Clinical Chemistry and Clinical Pharmacology, Bonn, Germany; The University of Hong Kong, Hong Kong

## Abstract

The preoperative differentiation between septic and aseptic loosening after total hip or knee arthroplasty is essential for successful therapy and relies in part on biomarkers. The objective of this study was to assess synovial and serum levels of inflammatory proteins as diagnostic tool for periprosthetic joint infection and compare their accuracy with standard tests. 120 patients presenting with a painful knee or hip endoprosthesis for surgical revision were included in this prospective trial. Blood samples and samples of intraoperatively acquired joint fluid aspirate were collected. White blood cell count, C-reactive protein, procalcitonin and interleukin-6 were determined. The joint aspirate was analyzed for total leukocyte count and IL-6. The definite diagnosis of PJI was determined on the basis of purulent synovial fluid, histopathology and microbiology. IL-6 in serum showed significantly higher values in the PJI group as compared to aseptic loosening and control, with specificity at 58.3% and a sensitivity of 79.5% at a cut-off value of 2.6 pg/ml. With a cut-off >6.6 pg/ml, the specificity increased to 88.3%. IL-6 in joint aspirate had, at a cut-off of >2100 pg/ml, a specificity of 85.7% and sensitivity of 59.4%. At levels >9000 pg/ml, specificity was almost at 100% with sensitivity just below 50%, so PJI could be considered proven with IL-6 levels above this threshold. Our data supports the published results on IL-6 as a biomarker in PJI. In our large prospective cohort of revision arthroplasty patients, the use of IL-6 in synovial fluid appears to be a more accurate marker than either the white blood cell count or the C-reactive protein level in serum for the detection of periprosthetic joint infection. On the basis of the results we recommend the use of the synovial fluid biomarker IL-6 for the diagnosis of periprosthetic joint infection following total hip and knee arthroplasty.

## Introduction

Periprosthetic joint infections (PJI) after total joint replacement are a severe complication and remain a key challenge in orthopedic surgery. Establishing a definite diagnosis of PJI prior to surgical intervention is at times difficult. However, the distinction between a periprosthetic joint infection and an aseptic loosening is crucial, as the treatment of aseptic loosening is completely different to the treatment of PJI [1]. The frequency of PJI is estimated to be 0.4–2% in primary total arthroplasty, rising up to 5–15% in high-risk patients and in revision surgery [2], [3], [4], [5]. A valid and standardized diagnostic procedure (clinical, laboratory, microbiological and histopathological findings) is essential to differentiate between PJI and aseptic loosening [3], [6], [7].

Analyzing the available evidence and existing published data on the definition and treatment of PJI a workgroup convened by the Infectious Diseases Society of America (IDSA) presented clinical practice guidelines including evidence-based and opinion-based recommendations for the diagnosis and management of PJI [8]. These recommendations are based on clinical findings, serum tests, histopathological results and synovial tests. Despite a significant amount of basic and clinical research in this field, there are still areas of controversy in which data are limited or conflicting. Consequently, there is a need for further research and development into new methods aimed at improving diagnostic accuracy and speed of detection.

Standard radiographs and laboratory blood analyses are used as first-line tests to determine the preoperative diagnosis of PJI. Although systemic inflammation markers such as the erythrocyte sedimentation rate, C-reactive protein (CRP) serum level, and white blood-cell count play a substantial role in the detection of PJI, they are not consistently reliable as they are highly sensitive but less specific [3], [9], [10]. The level of these nonspecific serum markers is affected by age, sex, and medical comorbidities of the patient. The white blood-cell count is rarely elevated in the presence of a chronic PJI and the CRP can be elevated for approximately 30 to 60 days in the immediate postoperative period limiting its predictive value [11], [12].

Recently published studies have suggested that interleukin-6 (IL-6) may be a helpful, and almost as accurate or even better marker for PJI as the CRP level or the erythrocyte sedimentation rate [13], [14]. Based on a meta-analysis Berbari et al. showed that serum IL-6 was associated with a high accuracy as a marker for PJI, followed by the CRP level, the erythrocyte sedimentation rate, and the white blood-cell count [11].

IL-6 is produced by monocytes and macrophages to stimulate immune response, and induces the production of major acute phase proteins, including CRP. Its role as anti-inflammatory cytokine is mediated through its inhibitory effects on TNF-alpha and IL-1 and activation of IL-1ra and IL-10. The serum IL-6 level in normal individuals is approximately 1 pg/mL, and it can increase to 30 to 430 pg/mL for as long as three days following total joint arthroplasty [14], [15]. Its serum level peaks two days after total joint arthroplasty, rapidly returns to normal value and is not elevated in patients with aseptic loosening [11], [16].

Deirmengian et al. previously measured protein biomarkers in synovial fluid that were significantly elevated in case of PJI [17]. Although these newer biomarkers seem to have better accuracy, their diagnostic utility has not been clearly established.

Following this rationale, and considering the need for a reliable marker in day-to-day clinical routine with high sensitivity and specificity for preoperative planning, we here present the IL-6 as a potential biomarker to differentiate between a painful total arthroplasty without signs of loosening and infection and aseptic loosened prosthesis on the one hand and PJI on the other hand.

Our hypothesis was that the concentration of IL-6 in serum and synovial fluid would align well with the presence or absence of a PJI and therefore could be of use in improving diagnostic accuracy and detection of a PJI. For this purpose we defined the sensitivity, specificity and accuracy of IL-6 in patients with PJI versus aseptic loosening and a control group and compared these results to current standards of diagnostic testing. Our null hypothesis was 1) that measured biomarkers in serum and synovial fluid do not correlate positively with the presence of PJI and 2) that the measured biomarkers are less useful than the current standards of diagnostic testing.

## Materials and Methods

The study was approved of by the local institutional review board (University on Bonn Ethics Committee). All patients signed informed consent prior to being enrolled in the study, and the study was conducted in accordance with the declaration of Helsinki. Between 2010 and 2011 we included 120 patients in our prospective study presenting with a painful total hip arthroplasty or total knee arthroplasty undergoing revision arthroplasty surgery for (1) PJI, (2) aseptic failure or (3) aseptic revision causes without signs of infection or loosening.

All patients underwent standardized diagnostics as outlined in the literature [18]. Pre operative blood and serum samples were collected from all patients. White blood cell count (WBC) was determined from the blood samples, and serum samples were analyzed for C reactive protein CRP (Dimension Vista, Siemens Medical Solutions Diagnostics GmbH, Eschborn, Germany), procalcitonin (PCT) (Immunoassay Analyzer Liaison (DiaSorin), Saluggia, Italy) and IL-6 (Immulite, Siemens Medical Solutions Diagnostics GmbH, Eschborn, Germany).

Joint aspiration was conducted under strictly aseptic conditions preoperatively or intraoperatively from the affected joint. The joint aspirate was incubated in aerobic, anaerobic, and fungal blood culture bottles as published [19]. The joint aspirate was analyzed cytologically according to descriptions by Trampuz et al. [20]. Synovial fluid was examined for total leukocyte count and cell differentiation by a blood count analyzer in body fluid mode as well as for IL-6 (Immulite, Siemens Medical Solutions Diagnostics GmbH, Eschborn, Germany) was measured within the aspirate.

At least, five periprosthetic tissue specimens were taken intraoperatively and divided into two single samples, one for the microbiological and one for the histopathological examination.

A PJI was considered proven if at least one of the following criteria was fulfilled (modified according to Parvizi et al. [21]):

1. Purulent synovial fluid or ≥ 1700 leukocytes/µl or ≥ 65% neutrophiles in the joint aspirate [TKA] (≥ 3600 leukocytes/µl or ≥ 80% neutrophiles [THA) [13]
2. Histological confirmation of a PJI (Typ II or Typ III according to the histopathological consensus classification of the periprosthetic interface by Morawietz et al. [22])
3. Pathogen detection in sterile joint aspiration or in at least two intra-operative tissue specimen after incubation
4. Definite signs of PJI clinically or intraoperatively (e.g. sinus tract) [6].

Those who did not meet these guidelines for a diagnosis of PJI and required a revision due to loosening were assigned as aseptic loosening (AL-group), those without signs of PJI nor loosening were assigned as controls (control group). Demographic data (age, sex, body mass index, type of prosthesis (THA/TKA) were collected for comparative analysis.

Data was collected in Microsoft Excel (Microsoft Corporation, Richmond, USA), and statistical analysis was carried out using GraphPad Prism 5.04 (GraphPad Software, La Jolla, CA, USA), testing for statistical significance between groups with one-way ANOVA without assuming normal distribution and Dunn’s post-hoc test. Receiver-Operator-Characteristiv (ROC) curves were used to asses discriminatory strength between PJI and non-PJI on the basis of area under the curve (AUC) and to determine optimal cut-off. Sensitivity and specificity for individual values and combinations were calculated.

## Results

We included a total of 120 patients into our prospective cohort study. The patient demographics and details are given in table 1. Though uneven in number, there was no statistical difference in age (p = 0.2686), patient gender (0.8611) or joint distribution (0.1110) between the three groups. More women than men were enrolled in the study, in concordance with other studies. In the PJI group and in the aseptic loosening group, more knee arthorplasties were recruited, while the control group had more hip arthroplasties, possibly due to the higher number of mechanical problems (dislocation & offset reconstruction) in hip arthroplasties, with a trend towards significance between aseptic and control group (p = 0.053).

**Table 1:** Patient Demographics.

Group	n total	Mean Age (+/− StdDev)	Sex (W:M)	Joint (Hip:Knee)
PJI	48	69.54 yrs (+/−12.14 yrs)	27 Female, 21 Male	22 TKA, 26 THA
Aseptic Loosening	51	68.04 yrs (+/−11.07 yrs)	33 Female. 18 Male	16 TKA, 35 THA
Control	21	64.05 yrs (+/−11.88 yrs)	13 Female, 8 Male	13 TKA, 8 THA
All	120	67.94 yrs (+/−11.71 yrs)	73 Female, 47 Male	51 TKA, 69 THA
P		0.2686	0.8611	0.1110

120 patients were enrolled in the study prospectively. Group assignment was done according to the criteria as mentioned above. There was no statistical difference in patient age, gender or distribution of joints in the groups. More women than men were enrolled in total and in all groups. There was a higher number of total hip arthroplasties (THA) than total knee arthroplasties (TKA) in the study population in all but the control group.

**Figure 1:**
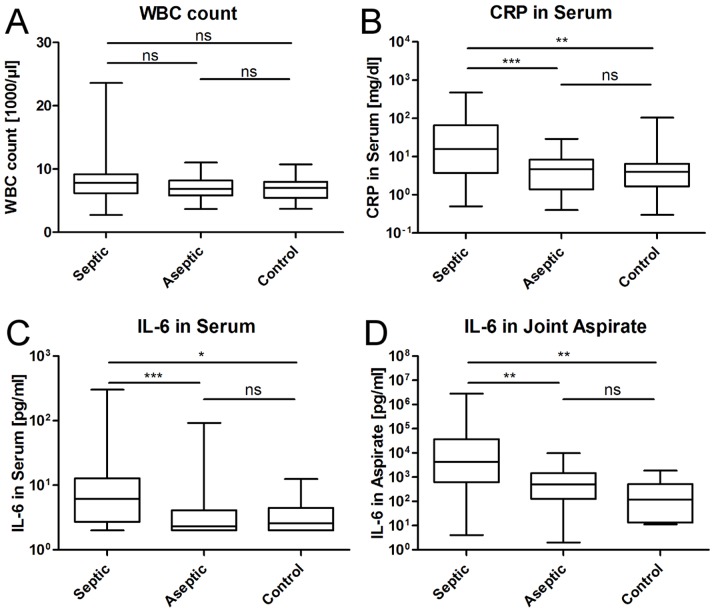
Differences in means between groups. Shown is the boxplot of the mean, 25%- and 75%-quartile and range of the values measured, as well as statistical differences between groups. Except for white blood cell count (A), all other parameters measured (B: CRP in Serum, C: IL-6 in Serum, D: IL-6 in joint aspirate) showed significant differences between the PJI group and the aseptic groups.

ANOVA with Dunn’s post hoc was completed to compare the means of laboratory values between the groups. The results are summarized in figure 1 A–D. The ANOVA for white blood cell count (WBC) showed no significant difference between the three groups. For the CRP levels in serum a significant difference between the three groups was seen, with p<0.0001. Post-hoc test confirmed significant differences between PJI and aseptic loosening (p<0.0001), as well as between PJI and control (p<0.001), but not between aseptic group and control. The same was true for PCT in serum, we found differences of the means between the groups (p<0.0001) with significant differences in septic vs. control (p<0.01) and septic vs. aseptic (p<0.001). IL-6 in serum and joint aspirate was considered as a potential marker to indicate PJI as mentioned above. In our analysis, IL-6 showed significantly higher values in the PJI group as compared to aseptic loosening (p<0.0001) and control (p<0.01). IL-6 in joint aspirate showed to be the most promising parameter measured, showing significant differences between PJI and the aseptic groups (both p<0.001), but again, not between aseptic loosening and control.

**Figure 2:**
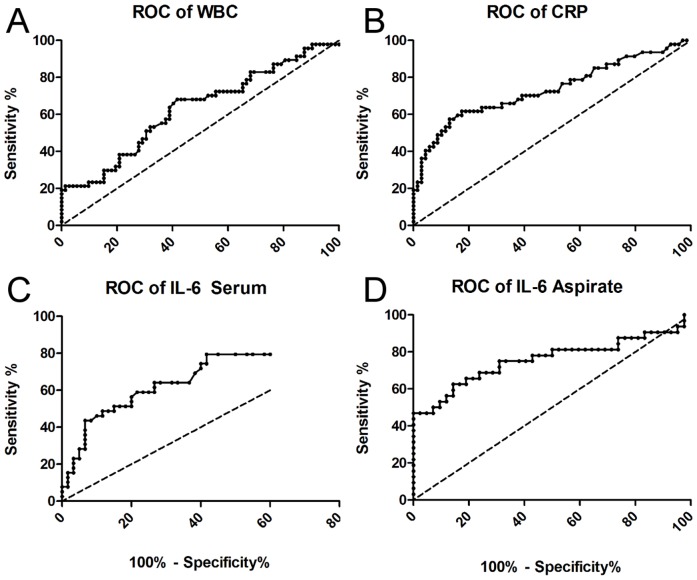
ROC curves of the markers measured. The curves A–D present the receiver-operator-characteristics curves of the parameters measured, depicting the area under the curve as an indicator for discriminatory strength. The line of identitiy is plotted as a dashed line in each graph.

To measure the discriminatory strength between PJI and non-PJI (aseptic loosening and control), we used receiver-operator-characteristics (ROC) curves (see Figure 2), measured the area under the ROC curves (AUC), defined the best cut-off values and calculated individual specificity and sensitivity. A summary of the results is given in table 2.

**Table 2:** Results summary.

Parameter	AUC (95% CI)	Cut-Off	Sensitivity (95% CI)	Specificity (95% CI)
WBC	0,63 (0,53 to 0,73)	10′300/µl	21,28% (10,70%–35,66%)	94,44% (86,38%–98,47%)
CRP	0,73 (0,64 to 0,83)	>9.1 mg/l	61,70% (46,38%–75,49%)	82,61 (71,59%–90,68%)
PCT	0,65 (0,51 to 0,80)	>46 ng/ml	12,90% (3,630%–29,83%)	100,0% (86,28%–100,0%)
IL-6 Serum	0,72 (0,61 to 0,83)	>2.6 pg/ml	79,49% (63,54%–90,70%)	58,33% (44,88%–70,93%
		>6.6 pg/ml	48,72% (32,42%–65,22%	88,33% (77,43%–95,18%)
IL-6 Joint Aspirate	0,76 (0,64 to 0,88)	>2100 pg/ml	62,50% (43,69%–78,90%)	85,71% (71,46%–94,57%
		>9000 pg/ml	46,88% (29,09%–65,26%)	97,62% (87,43%–99,94%)

Optial cut-off values were determined and individual sensitivity and specificity was calculated for each marker to identify a periprosthetic joint infection. IL-6 in joint aspirate showed to be the most promising candidate to indicate a PJI.

White blood cell count (WBC) showed a specificity of 94.5% (95% CI: 86.4% to 98.5%) for PJI at a cut-off level of 10′300 Leukocytes/µl with a likelihood ratio of 3.83, but a poor sensitivity of just 21.3%. The AUC equaled 0.63 (95% CI: 0.52 to 0.73, p>0.01). 20% of the patients with PJI presented with an increased leukocyte count in peripheral blood. The specificity for CRP to detect a PJI was 82.6% with a sensitivity 61.7% at a cut-off value of >9 mg/dl. The area under the curve of the ROC equaled 0.73 (95% CI: 0.63 to 0.83, P<0.0001). The likelihood ratio was 3.55.

For procalcitonin (PCT) in serum, the cut-off value is commonly set at 46 ng/l. The sensitivity here was at 12.9%, even below that of white blood cell count with 21.3%. In addition, patients with elevated PCT levels already presented with signs of SIRS or sepsis and all other signs of fulminant purulent infection.

IL-6 in serum showed a specificity at 58.3% with a sensitivity of 79.5% at a cut-off value of >2.6 pg/ml. With a cut-off above 6.6 pg/ml the specificity increased to 88.3% with a sensitivity of 48%. The area under the curve of the ROC equaled 0.72 (95% CI: 0.612 to 0.828, p = 0,0002). Cut-off values for IL-6 in synovial fluid are varying and not yet well defined in the literature. We found that at a cut-off of more than 2100 pg/ml, specificity was at 85.7% and sensitivity at 59.4%. The area under the curve in the ROC was 0.76 (0.635 to 0.88, p = 0,0001). At levels >9000 pg/ml, specificity was almost at 100% with sensitivity just below 50%, so PJI could be considered proven with IL-6 levels above this threshold.

We calculated if a combination of IL-6 in serum and aspirate could enhance predictive power over singe values. Cut-off was defined for IL-6 in serum at 2.6 pg/ml and for IL-6 in aspirate at 2100 pg/ml. The positive prediction value for a PJI with IL-6 positive in both serum and aspirate was at 0.89, the negative prediction value with both negative at 0.78. However, with one out of two positive, results are difficult to interpret, with ppv and npv almost coming equal (ppv: 0.547, npv: 0.453).

## Discussion

Accurate preoperative identification of PJI is difficult, as the clinical symptoms often resemble those of aseptic loosening, with nonspecific pain. To prevent unnecessary two-stage procedures in case of false-positive diagnosis of PJI correct preoperative diagnosis is important. Furthermore, failure to diagnose PJI prior to revision surgery would result in one-stage revision without appropriate treatment, most likely resulting in persisting infection and recurrent implant failure. By the number of studies attempting to identify the best combination of laboratory tests predicting PJI, the recent literature has highlighted the need for improved diagnostics [23], [24], [25], [26].

The erythrocyte sedimentation rate and serum level of CRP are, with reported accuracies of 0.75 and 0.81, respectively, sensitive but less specific markers for the diagnosis of PJI [11]. Furthermore, because systemic tests for PJI would be misconceived by concomitant infection at another anatomic site or any systemic inflammatory disease, a synovial fluid test for PJI is intuitively attractive. Parvizi et al. demonstrated recently, that the measurement of CRP levels in synovial fluid rather than the serum increases the diagnostic accuracy in identifying PJI, thus holding great promise as a synovial biomarker to distinguish PJI from aseptic loosening Nevertheless, despite the best efforts, some patients will remain undiagnosed until the time of surgery.

Interleukin-6 (IL-6) is a 26-kilodalton pleiotropic cytokine that functions as pro- and anti-inflammatory molecule, promoter of hematopoiesis and as inducer of plasma-cell development [27]. IL-6 is produced by monocytes and macrophages to stimulate immune response and is one of the most important mediators of fever and acute phase response [28]. Serum IL-6 has been shown to be a valuable and even more accurate marker than either the erythrocyte sedimentation rate or the CRP level for the detection of chronic PJI [15]. In a relatively small study, the combination of CRP and IL-6 identified all patients with PJI and showed a specificity of 1.00 (0.99–1.00) with high positive and negative predictive values [16]. Disadvantages of serum tests are that they are nonspecific and may increase in response to several diseases with acute inflammatory reactions. Limitations of the IL-6 diagnostic method in serum are the reportedly elevated IL-6 levels in patients with chronic inflammatory diseases, Paget disease and immunodeficiency syndromes. This could be proven by the results of the present study, which demonstrated significantly higher serum levels for IL-6 for PJI compared to aseptic loosening and the control group, with a specificity of 58.3% and an area under the curve of the 0.72.

Deirmengian et al. identified twelve synovial fluid biomarkers with substantially higher average levels in the synovial fluid of infected versus aseptic patients, among them, IL-1, IL-6, IL-17, and G-CSF with a high accuracy [17]. The authors were able to demonstrate that IL-1 and IL-6 were significantly increased in synovial fluids from patients with PJI compared with patients without infection, with an AUC of above 0.9. With regard to diagnostic accuracy, the data presented by Deirmengian et al. indicate that synovial biomarkers have the potential to outperform established predictors of PJI. Similar promising results showed a study by Gollwitzer et al. assessing intra-articular and systemic levels of antimicrobial peptides and pro-inflammatory cytokines as diagnostic markers for PJI [28].

The purpose of the current study was to evaluate IL-6 as synovial fluid biomarker for PJI, and compare its diagnostic characteristics to current standard laboratory tests. We emphasize, we did not exclude patients with inflammatory disease or patients under current antibiotic therapy.

Our results are in accordance with recent literature showing significant local increase of IL-6 in synovial fluid as well as systemic upregulation of IL-6 in serum [14], [17], [29], [30]. The present study demonstrates that the synovial level of IL-6 appears to be a more accurate marker than either the C-reactive protein level or the IL-6 level in serum for the detection of PJI. Correct detection of infection is essential and critical in the diagnostics. A false negative result with a missed infection can have catastrophic consequences for the patient with persistence or recurrence of infection. IL-6 can enhance the detection of a PJI significantly and add to the canon of diagnostic tools already present. On the basis of these results we recommend the use of serum and synovial IL-6 level in combination with standard diagnostic evaluation, to test for PJI following total hip and knee arthroplasty. At serum IL-6 levels >2.6 pg/ml or synovial fluid levels >2100 pg/ml, a PJI should be considered very likely, and >6.6 pg/ml (or >9000 pg/ml, respectively) considered proven.

We acknowledge that our study has limitations. The sample size is low for a study investigating arthroplasties, even though the number of patients is high for the treatment of PJI in infected TKAs and THAs. The inhomogenity of the patients investigated is both a weakness and strength of the paper. Patients with PJI are complex and difficult to compare, but this represents day-to-day clinical experience. Eventually, new biomarkers and a further modification of the published therapy algorithm may become necessary.

## References

[1] Johnson AJ, Zywiel MG, Stroh A, Marker DR, Mont MA (2011) Serological markers can lead to false negative diagnoses of periprosthetic infections following total knee arthroplasty. Int Orthop 35: 1621–1626. 21181540 10.1007/s00264-010-1175-5 PMC3193961

[2] Segawa H, Tsukayama DT, Kyle RF, Becker DA, Gustilo RB (1999) Infection after total knee arthroplasty. A retrospective study of the treatment of eighty-one infections. J Bone Joint Surg Am 81: 1434–1445. 10535593 10.2106/00004623-199910000-00008

[3] Zimmerli W, Trampuz A, Ochsner PE (2004) Prosthetic-joint infections. N Engl J Med 351: 1645–1654. 15483283 10.1056/NEJMra040181

[4] Jamsen E, Huhtala H, Puolakka T, Moilanen T (2009) Risk factors for infection after knee arthroplasty. A register-based analysis of 43,149 cases. J Bone Joint Surg Am 91: 38–47. 10.2106/JBJS.G.01686 19122077

[5] Peersman G, Laskin R, Davis J, Peterson M (2001) Infection in total knee replacement: a retrospective review of 6489 total knee replacements. Clin Orthop Relat Res: 15–23. 11716377

[6] Trampuz A, Zimmerli W (2005) New strategies for the treatment of infections associated with prosthetic joints. Curr Opin Investig Drugs 6: 185–190. 15751742

[7] Frommelt L (2004) [Guidelines on antimicrobial therapy in situations of periprosthetic THR infection]. Orthopade 33: 822–828. 15156312 10.1007/s00132-004-0677-5

[8] Osmon DR, Berbari EF, Berendt AR, Lew D, Zimmerli W, et al (2013) Diagnosis and management of prosthetic joint infection: clinical practice guidelines by the Infectious Diseases Society of America. Clin Infect Dis 56: e1–e25. 23223583 10.1093/cid/cis803

[9] Ghanem E, Antoci V Jr, Pulido L, Joshi A, Hozack W, et al (2009) The use of receiver operating characteristics analysis in determining erythrocyte sedimentation rate and C-reactive protein levels in diagnosing periprosthetic infection prior to revision total hip arthroplasty. Int J Infect Dis 13: e444–449. 19473865 10.1016/j.ijid.2009.02.017

[10] Parvizi J, Ghanem E, Menashe S, Barrack RL, Bauer TW (2006) Periprosthetic infection: what are the diagnostic challenges? J Bone Joint Surg Am 88 Suppl 4138–147. 10.2106/JBJS.F.00609 17142443

[11] Berbari E, Mabry T, Tsaras G, Spangehl M, Erwin PJ, et al (2010) Inflammatory blood laboratory levels as markers of prosthetic joint infection: a systematic review and meta-analysis. J Bone Joint Surg Am 92: 2102–2109. 20810860 10.2106/JBJS.I.01199

[12] Toossi N, Adeli B, Rasouli MR, Huang R, Parvizi J (2012) Serum white blood cell count and differential do not have a role in the diagnosis of periprosthetic joint infection. J Arthroplasty 27: 51–54 e51. 10.1016/j.arth.2012.03.021 22608690

[13] Glehr M, Friesenbichler J, Hofmann G, Bernhardt GA, Zacherl M, et al (2013) Novel biomarkers to detect infection in revision hip and knee arthroplasties. Clin Orthop Relat Res 471: 2621–2628. 23609811 10.1007/s11999-013-2998-3 PMC3705066

[14] Bottner F, Wegner A, Winkelmann W, Becker K, Erren M, et al (2007) Interleukin-6, procalcitonin and TNF-alpha: markers of peri-prosthetic infection following total joint replacement. J Bone Joint Surg Br 89: 94–99. 17259424 10.1302/0301-620X.89B1.17485

[15] Di Cesare PE, Chang E, Preston CF, Liu CJ (2005) Serum interleukin-6 as a marker of periprosthetic infection following total hip and knee arthroplasty. J Bone Joint Surg Am 87: 1921–1927. 16140805 10.2106/JBJS.D.01803

[16] Buttaro MA, Tanoira I, Comba F, Piccaluga F (2010) Combining C-reactive protein and interleukin-6 may be useful to detect periprosthetic hip infection. Clin Orthop Relat Res 468: 3263–3267. 20623261 10.1007/s11999-010-1451-0 PMC2974855

[17] Deirmengian C, Hallab N, Tarabishy A, Della Valle C, Jacobs JJ, et al (2013) Synovial fluid biomarkers for periprosthetic infection. Clin Orthop Relat Res 468: 2017–2023. 10.1007/s11999-010-1298-4 PMC2895851 20300901

[18] Wimmer MD, Randau TM, Petersdorf S, Pagenstert GI, Weisskopf M, et al.. (2013) Evaluation of an interdisciplinary therapy algorithm in patients with prosthetic joint infections. Int Orthop. 10.1007/s00264-013-1995-1 PMC3824888 23851647

[19] Weiss S, Geiss H, Kommerell M, Simank HG, Bernd L, et al.. (2006) [Improving the diagnosis of septic arthritis by use of a pediatric blood culture system]. Orthopade 35: 456, 458–462. 10.1007/s00132-005-0900-z 16344955

[20] Trampuz A, Hanssen AD, Osmon DR, Mandrekar J, Steckelberg JM, et al (2004) Synovial fluid leukocyte count and differential for the diagnosis of prosthetic knee infection. Am J Med 117: 556–562. 15465503 10.1016/j.amjmed.2004.06.022

[21] Parvizi J, Zmistowski B, Berbari EF, Bauer TW, Springer BD, et al (2011) New definition for periprosthetic joint infection: from the Workgroup of the Musculoskeletal Infection Society. Clin Orthop Relat Res 469: 2992–2994. 21938532 10.1007/s11999-011-2102-9 PMC3183178

[22] Morawietz L, Classen RA, Schroder JH, Dynybil C, Perka C, et al (2006) Proposal for a histopathological consensus classification of the periprosthetic interface membrane. J Clin Pathol 59: 591–597. 16731601 10.1136/jcp.2005.027458 PMC1860400

[23] Bare J, MacDonald SJ, Bourne RB (2006) Preoperative evaluations in revision total knee arthroplasty. Clin Orthop Relat Res 446: 40–44. 16672870 10.1097/01.blo.0000218727.14097.d5

[24] Della Valle CJ, Sporer SM, Jacobs JJ, Berger RA, Rosenberg AG, et al (2007) Preoperative testing for sepsis before revision total knee arthroplasty. J Arthroplasty 22: 90–93. 17823024 10.1016/j.arth.2007.04.013

[25] Austin MS, Ghanem E, Joshi A, Lindsay A, Parvizi J (2008) A simple, cost-effective screening protocol to rule out periprosthetic infection. J Arthroplasty 23: 65–68. 18165031 10.1016/j.arth.2007.09.005

[26] Schinsky MF, Della Valle CJ, Sporer SM, Paprosky WG (2008) Perioperative testing for joint infection in patients undergoing revision total hip arthroplasty. J Bone Joint Surg Am 90: 1869–1875. 18762646 10.2106/JBJS.G.01255

[27] Nishimoto N Interleukin-6 as a therapeutic target in candidate inflammatory diseases. Clin Pharmacol Ther 87: 483–487. 20182422 10.1038/clpt.2009.313

[28] Mihara M, Hashizume M, Yoshida H, Suzuki M, Shiina M IL-6/IL-6 receptor system and its role in physiological and pathological conditions. Clin Sci (Lond) 122: 143–159. 22029668 10.1042/CS20110340

[29] Nilsdotter-Augustinsson A, Briheim G, Herder A, Ljunghusen O, Wahlstrom O, et al (2007) Inflammatory response in 85 patients with loosened hip prostheses: a prospective study comparing inflammatory markers in patients with aseptic and septic prosthetic loosening. Acta Orthop 78: 629–639. 17966022 10.1080/17453670710014329

[30] Gollwitzer H, Dombrowski Y, Prodinger PM, Peric M, Summer B, et al. Antimicrobial peptides and proinflammatory cytokines in periprosthetic joint infection. J Bone Joint Surg Am 95: 644–651. 23553300 10.2106/JBJS.L.00205

